# Editorial: Erythrocytes as a target of oxidative stress in blood

**DOI:** 10.3389/fphys.2023.1310053

**Published:** 2023-11-07

**Authors:** Alessia Remigante, Elisabetta Straface, Angelo D’Alessandro, Rossana Morabito

**Affiliations:** ^1^ Department of Chemical, Pharmaceutical and Environmental Sciences, University of Messina, Messina, Italy; ^2^ Center for Gender-Specific Medicine, Istituto Superiore di Sanità, Rome, Italy; ^3^ Department of Biochemistry and Molecular Genetics, University of Colorado Anschutz Medical Campus, Aurora, CO, United States

**Keywords:** red blood cells, Erythrocytes, physiology, redox, oxidative stress

Red blood cells (RBCs) are unique, highly specialized and the most abundant cells in different organisms. Although their primary function is the transport of respiratory gases from lungs to tissues and back, these cells are equipped with effective anti-oxidative systems that make them circulating free radical scavengers, thus providing antioxidant protection not only to themselves but also to other tissues and organs in the body. However, excess levels of oxidative stress (OS) have deleterious effects on cell components and alter the redox status of the RBC (Remigante et al., 2022a; D'Alessandro et al., 2023). OS triggers biochemical changes in human RBCs (e.g., oxidation of key amino acid residues in metabolic enzymes or the disruption in the molecular arrangement of the bilayer), as well as biophysical alterations (e.g., changes in the general structural arrangement, morphology and mechanics (Remigante et al., 2022b; Spinelli et al., 2023)). The aim of this Research Topic was to gather a of studies that, while incomplete, would contribute to advancing our understanding of the RBC response to OS, by focusing on the investigation of the molecular mechanisms underlying the effects of OS on RBCs and their cellular components. Hereafter we offer an overview of the content of the Research Topic.

RBCs deformability is exceptional among mammalian cells and facilitates nutrient delivery throughout the microcirculation. However, this physical property is negatively impacted by OS. Kuck et al. explored both the effects of intracellular superoxide (O_2_^−^) generation on the RBC deformability and the activation of pivotal molecular pathways known to regulate cell mechanics. RBC exposed to O_2_^−^ were conditioned with specific shear stresses, prior to evaluation of cellular deformability and activation of PI3K/Akt kinase and RBC-NOS (nitric oxide synthase). Intracellular generation of O_2_^−^ decreased phosphorylation of RBC-NOS as well as phosphorylation of Akt kinase was also diminished. Impaired RBC deformability induced by intracellular O_2_^−^ may be due, in part, to impaired activation of PI3K/Akt, and downstream signalling with RBC-NOS. These results may shed light on improved circulatory health with targeted promotion of blood flow, and may prove fruitful in future development of blood-contacting devices.

Rheumatoid arthritis (RA) is a chronic autoimmune disease associated with a significantly increased risk of cardiovascular mortality with a prevalence in women. The purpose of this investigation, performed by Di Franco et al. was to correlate the RA disease to RBC functional characteristics. Disease activity, measured as the number of swollen and painful joints (DAS-28), was correlated with a) estrogen receptor expression, which modulates the intracellular signalling; b) activation of the estrogen-linked kinase ERK1⁄2, which is a key regulator of RBC adhesion and survival; and c) levels of inflammatory and OS-related biomarkers. Each biomarker was evaluated in RA patients at baseline and 6 months after treatment with disease-modifying anti-rheumatic drugs (DMARDs). Data suggest that in RA patients a) the DAS-28 correlated with RBC ER-α expression, and did not correlate with total antioxidant capacity of plasma; b) the RBC ER-α expression correlated with systemic inflammatory biomarkers and OS parameters, as well as ERK1⁄2 phosphorylation; c) treatments with DMARDs improved the clinical condition measured by DAS-28 score decrease, although the RBCs appeared to be more prone to pro-oxidant status associated to the survival molecule expression. These data represent an important advance in the study of RA determinants favouring the developing of cardiovascular diseases, because strongly suggest that RBCs could also participate in the vascular homeostasis through fine modulation of an intracellular signal linked to the ER-α.

Sickle cell disease is a genetic blood disorder. Sickle cell haemoglobin (HbS), when oxidized with H_2_O_2_, stays longer in a highly oxidizing ferryl (Fe^4+^) form causing irreversible oxidation of βCys93 to a destabilizing cysteic acid. Kassa et al. have reported that an anti-sickling drug can be designed to bind specifically to βCys93 and protect against its irreversible oxidation by H_2_O_2_. Here, oxygen dissociation, oxidation, and polymerization kinetic reactions for four anti-sickling drugs that either site-specifically target βCys93 or other sites on the HbS molecule are reported. Molecules that specifically bind to or modify βCys93, such as 4,4′-di (1,2,3-triazolyl) disulphide (TD-3) and hydroxyurea (HU) were contrasted with molecules that target other sites on Hb including 5-hydroxymethyl-2-furfural (5-HMF) and L-glutamine. All reagents induced a left shift in the oxygen dissociation curve except L-glutamine. In the H_2_O_2_ presence both TD-3 and HU reduced the ferryl heme by 22% and 37%, respectively, which corresponded to a 3- to 2-fold reduction in the levels of βCys93 oxidation as verified by mass spectrometry. Increases in the delay times prior to polymerization of HbS under hypoxia were in the following order: TD-3>HU > 5-HMF = L-glutamine. Designing anti-sickling agents that can specifically target βCys93 may provide a dual antioxidant and anti-sickling therapeutic benefits in treating this disease.

Exercise intolerance is a common clinical manifestation in patients with sickle cell disease. Cendali et al. leverage a murine mouse model of sickle cell disease, the Berkeley mouse, to characterize response to exercise via determination of critical speed (CS), a functional measurement of mouse running speed upon exerting to exhaustion. After observing a wide distribution in critical speed phenotypes, they systematically determined metabolic aberrations in plasma and some organs from mice ranked based on critical speed performances. Results indicated clear signatures of systemic and organ-specific alterations in carboxylic acids (especially succinate), sphingosine 1-phosphate and acylcarnitine metabolism. Metabolites in these pathways showed significant correlations with critical speed across all matrices. Findings from murine models were thus further validated in 433 sickle cell disease patients. Metabolomics analyses of plasma from 281 subjects in the WALK PhASST cohort were used to identify metabolic correlates to sub-maximal exercise test performances, as measure by 6 min walking test in this clinical cohort. Results confirmed strong correlation between test performances and dysregulated levels of circulating succinate and sphingosine 1-phosphate. Novel circulating metabolic markers of exercise intolerance in mouse models of sickle cell disease and sickle cell patients have been identified.

To conclude, the publications collected are well representative of the variety of roles of RBCs and highlight the multitude of the experimental models and approaches currently used to unravel their complexity. While acknowledging the limited scope of this Research Topic, we hope that this editorial effort will contribute to fuelling the burgeoning field of RBC biochemical and biophysical responses to oxidant stress, an area we believe to be relevant not only to haematology and transfusion medicine, but to human physiology and medical investigations at large.
